# The Cure of Fistula in Ano without Operation

**Published:** 1887-07

**Authors:** E. Andrews

**Affiliations:** Chicago, Ill.


### THE CURE OF FISTULA IN ANO WITHOUT OPERATION.

BY E. ANDREWS, M. D., L.L.D., CHICAGO, ILL.

WHILE preparing the materials for a work on "Rectal Surgery" some interesting facts come to my knowledge. The general impression among physicians is that nothing will cure a fistula except a surgical operation; and, indeed, this is true with regard to many fistulas. But it is equally true that a large proportion of them are curable without any operation more serious than probing and injecting, and in such cases it is a duty to exhaust the milder measures before resorting to the severer. But it is necessary to make a proper selection of cases. Where the fistula leads to extensive pouches, or several complicated branches leading in different directions about the rectum, the non-operative methods are not likely to succeed in any moderate length of time. But where the fistula is simple and contains no large pouches, and leads pretty directly to an opening in the rectum, there is an excellent prospect of cure without any strictly operative procedures. The reason why a stricture does not cure itself is not altogether, as we have formerly supposed, the daily forcing through it of materials from the rectum. On the contrary, if the interior of the fistula be thoroughly antiseptisized throughout its entire length and into every curve and corner, and maintained in that purified condition, the ulcerative tendency which prevents contraction and healing is arrested. Granulations spring up through the whole length of the passage, and close it in spite of any moderate tendency of the rectum to force mucus, gas and fæces into its channel.

Naturally enough, the itinerant quacks who are traversing every part of the Mississippi Valley—and, for the most part, are so unskilled that they do not dare undertake a cutting operation—have been among the first to discover the success of the non-operative methods, though they are too ignorant to understand the principle on which they accomplish their cures. For instance, a man named Brinkerhoff, in Ohio, sells to itinerants for a price varying from $100 to $300, according to the gullibility of the purchaser, a little secret book of directions, constituting what is sometimes called the "Brinkerhoft System of Rectal Surgery." With this book of directions goes a little case containing about twelve dollars' worth ot instruments, among which there is nothing by which an itinerant could possibly make an incision. He is too ignorant and cannot be trusted with edged tools; and, in fact, in his little book, Brinkerhoff says, in regard to fistula: "Never use the knife or ligatures." He directs the itinerant first to oil the interior of the rectum and the external integuments around. Next he is to inject the fistula thoroughly with an antiseptic mixture, and, taking a probe, churn the medicine thoroughly through every part of the fistula, and then, closing the external orifice with one finger, he is with the other finger to compress the external parts of the channel so as to compel the antiseptic to run thoroughly into the deeper parts. This accomplished he injects 10 or 15 drops of the following mixture:

℞ Distilled extract witch hazel........fl ʒiii

Liquor of the persulphate of iron..fl ʒi

Carbolic acid........................grs ij

Glycerine............................fl ʒij

Mx.

The itinerant is then to apply an antiseptic mixture to the internal orifice of the fistula and to any ulcers in its vicinity. He is to repeat this treatment every two or four weeks, depending not on the welfare of the patient, but upon whether the quack is working a two-weeks' or a four-weeks' circuit. Other irregulars take the following course: First having explored the fistula well with a flexible probe, they wash out its channel with a solution of hydrogen peroxide. They then take equal parts of 95 per cent. carbolic acid and a 10 per cent. solution of cocaine and inject carefully but thoroughly 10 to 15 minims of the mixture into the fistula. The patient is then to lie down about two hours, when he receives into the fistula an injection of equal parts of *Ol. eucalypti* and glycerin, with orders to keep quiet for two days more. These men have discovered that many fistulas can be cured by such antiseptic methods, although they generally do not understand the principle on which it is accomplished. Their ignorance of general science, however, need not deter us from observing the results of their experiments, and applying the principles involved in them for the benefit of our patients. Dr. Matthews, a regular physician of Louisville, has devised a treatment which may almost be called non-operative and by which he claims to have cured about twenty cases. His plan is the following: He takes a long slender laminaria tent, and, guiding it into the fistula as far as it will go, leaves it several hours to dilate the passage. He then takes Otis' curethrotome, and, insinuating it into the sinus, turns the screw and moderately dilates the channel, after which, by protruding the knife concealed in the tip of the instrument, he scarifies the interior. If necessary, he repeats the operation several times, thus making the passage straight and simple, giving it a free external opening, and by the irritation of the incisions arousing the growth of new granulations. If one wishes to try the completely non-operative plan, the best method of procedure is as follows: First, explore the interior and ascertain that it is simple enough to give the prospect of being able to make the injections reach all parts of it. Next, bear in mind that this fistula must have a free external opening, otherwise it will confine a quantity of septic pus in the interior, which, both by mechanical distension and irritant qualities, will arrest all efforts at healing. It is therefore best in many case to enlarge the external portion of the sinus with a bistoury or with a laminaria tent. This being accomplished, inject the whole interior of the fistula carefully with a good vigorous article (for that sold in the shops is very variable) of hydrogen peroxide. It will be better to inject this through a small catheter inserted into the deepest parts of the channel, or else throw it in by a syringe whose beak is large enough to completely fill the fistulous opening, so that the pressure shall compel the fluid to find its way into the remotests parts. Throwing the solution in repeatedly, and giving it time between the pulsations of the syringe for the foam produced by the action of the medicine on the pus to boil freely out, we next leave the patient quietly on the lounge an hour or two. Then with a small syringe insert about 10 minims of a solution of bichloride ot mercury of the strength of one part to 3,000 of water. Repeat this once in about 3 or 5 days, taking pains not to threw large quantities of irritating solutions through into the rectum. This procedure alone will cure a considerable proportion of cases. But if greater thoroughness is required, some advantage will be gained by taking Allingham's rectal speculum and, exposing the internal orifice of the fistula, touch the opening with a stick of nitrate of silver where it enters the rectum, and place the patient in bed with a sheaf of three soft rubber catheters lying side by side in the rectum to give exit to the gases and mucus. The bowels should be previously emptied with a cathartic.

There has been a very general opinion in the profession that it is not expedient to cure a fistula where the patient is inclined to tuberculosis. Dr. E. E. Glover, of Terre Haute, Ind., has taken the pains to ascertain of a large number of surgeons, on both continents, their opinion on this subject, by which he discovers that there is apparently a very great change of opinion on this question. He finds that those who reply to the question as to whether they would operate in tuberculous cases the following who say yes: Allingham, Agnew, Andrews, Brinton, Brodie, Borck, Bonteson, Solis-Cohen, Cole, Francis Delafield, Eastman, Englemann, Gunn, Hamilton, E. F. Ingals, Lane, Linthicum, McGuire, Mathews, Moore, Owens. Prewitt, Peck, Raemy, Sayre, T. G. Richardson, of New Orleans, Roberts, of Philadelphia, Wight, Wilson, Varich and Taylor.

My own opinion is that there is no objection to curing the fistula in such cases. On the contrary, it is beneficial to the patient to do so. But it is true that where it is done by incision the wounds do not always heal well, and if the patient has but a year or two to live on account of his tuberculosis it seems scarcely worth while to submit to the annoyance of the operation. But this would be no reason why he might not be advantageously treated by gentle and non-operative methods, such as we have described.

